# Development of nanocomposite-selenium filter for water disinfection and bioremediation of wastewater from Hg and AgNPs

**DOI:** 10.1038/s41598-024-70120-3

**Published:** 2024-09-13

**Authors:** Osama M. Darwesh, Ibrahim A. Matter, Mostafa A. Abdel-Maksoud, Wahidah H. Al-Qahtani, Mohamed A. El-Tayeb, Ahmad S. Kodous, Mohammed Aufy

**Affiliations:** 1 Agricultural Microbiology Department, National Research Centre, 33 El-Buhouth St., Dokki, Cairo 12622 Egypt; 2 Botany and Microbiology Department, College of Science, King Saud University, Riyadh, Saudi Arabia; 3 Department of Food Sciences and Nutrition, College of Food and Agricultural Sciences, King Saud University, P.O. Box 270677, 11352 Riyadh, Saudi Arabia; 4 Department of Molecular Oncology, Cancer Institute (WIA), 38, Sardar Patel Road, P.O. Box 600036, Chennai, Tamilnadu India; 5 Department of Pharmaceutical Sciences, Division of Pharmacology and Toxicology, University of Vienna, Vienna, Austria

**Keywords:** SeNPs biosynthesis, *Bacillus pumilus*, Nanochitosan, Polysaccharide, Heavy metals removal, SeNPs/NCh biofilter, Biological techniques, Biotechnology, Microbiology, Environmental sciences

## Abstract

Selenium nanoparticles (SeNPs) are used in several sectors as antitumor, antimicrobial, and environmental adsorbents. Thus, the present research objective was the production of bacterial-SeNPs as an active and environmentally-friendly antibacterial and adsorbent agents and application into novel nanocomposite filter. From a total of 25 samples (soil, wastewater, and water) obtained from different locations in Egypt, 60 selenium-resistant bacterial isolates were obtained (on a mineral salt medium supplemented with selenium ions). After screening (based on the conversion of selenium from ionic form to nanoform), a superior bacterial isolate for SeNPs formation was obtained and molecular identified as *Bacillus pumilus* isolate OR431753. The high yield of SeNPs was noted after optimization (glucose as carbon source, pH 9 at 30 °C). The produced SeNPs were characterized as approximately 15 nm-diameter spherical nanoparticles, in addition to the presence of organic substances around these particles like polysaccharides and aromatic amines (protein residues). Also, they have antibacterial activity increased after formation of nanocomposite with nano-chitosan (SeNPs/NCh) against several pathogens. The antibacterial activity (expressed as a diameter of the inhibitory zone) averaged between 2.1 and 4.3, 2.7 and 4.8 cm for SeNPs and SeNPs/NCh, respectively compared with 1.1 to 1.8 cm for Amoxicillin. The produced nanoselenium/chitosan was used as a biofilter to remove mercury (Hg) and AgNPs as model chemicals with serious toxicity and potential pollutant for water bodies in many industries. The new SeNPs/NCh biofilter has proven highly effective in individually removing mercury and AgNPs from their synthetic wastewaters, with an efficiency of up to 99%. Moreover, the removal efficiency of AgNPs stabilized at 99% after treating them with the syringe filter-Se nanocomposite for 4 cycles of treatment (5 min each).

## Introduction

As a result of urban and industrial expansion, many organic and inorganic pollutants are discharged into various water sources daily, negatively affecting the aquatic environment, animals, and human health. Unlike organic pollutants, inorganic pollutants do not decompose, so they must be appropriately removed from contaminated aquatic environments. Mercury and silver are toxic heavy metals of major environmental concern because of their health effects on biological systems and their ability to accumulate widely in the environment^1^. Mercury is a highly toxic substance that is found in most bodies of water, especially those near human industrial activities including coal burning, oil and natural gas refining, household waste burning, and mining^2^. Silver is also considered a nuisance pollutant in aquatic environments due to its harmful effects on mammalian cells and aquatic species. Although silver is less toxic than mercury, concerns about silver contamination are increasing, especially in light of the increasing number of applications based on nanosilver^3^. Removing such toxic elements (and other pollutants) from various aquatic environments is of great environmental and health importance, which calls for more innovative scientific efforts to solve it. In this regard, many physical, chemical, and biological methods have been developed to remove Hg^2+^, Ag ions, and Ag-nanoparticles (AgNPs) from contaminated water bodies^4,5^.

The removal of Hg^2+^, and Ag ions, (both ionic and nanoforms) and other heavy metals from wastewater can be performed using a variety of efficient physical, chemical, and biological techniques. Physical and chemical processes consist of foam flotation, coagulation, magnetic separation, solvent extraction, electrochemical precipitation, mechanical screening, membrane filtration, chemical precipitation, and activated carbon adsorption. These conventional methods' high costs, poor selectivity, adverse effects from organic matter, inefficiency at low metal concentrations, and significant reagent and energy requirements make them unattractive^5^. Recently, there has been a move to apply the use of nanomaterials technology to remove such a toxic element (and other pollutants) from aquatic environments. In this regard, a notable number of nanomaterials have been developed for Hg^2+^ and AgNPs removal from water bodies^5,6^. Selenium is a crucial trace element that the human body needs in a small quantity (up to 40–300 µg daily) to maintain its vital functions^7^. It helps safeguard cardiovascular health, control thyroid hormones, stimulate the immune system, and stop the spread and growth of cancer cells^8^. Selenium nanoparticles are high active and less toxic than another forms and used in several applications as antitumor^9^, antimicrobial^10^ and drug delivery. The selenium nanoparticle has a wide average size, and its characteristics and biological activity are affected by the biomolecule's kind, size, and shape. The biological activity is strengthened by the selenium nanoparticles' large surface area and small particle size^11^. Selenium in particular can bind with mercury with high binding force and can therefore be exploited for its removal from aquatic environments, if the selenium is immobilized on a suitable material. In addition, it can be applied to remove any heavy metals from polluted wastewater, especially when selenium is in a complex form with another catalytic compound such as ZnO/Se nanocomposite^12^. Zambonino and coworkers reported the success of using selenium in the form of SeNPs to effectively remove mercury up to 100 times higher than commercially available sorbents including activated carbon^13^. Moreover, the natural polymer Chitosan is rich in amino (-NH_2_) and hydroxyl (-OH) groups, giving it strong adsorption capacity and reactivity with most pollutants. Thus, chitosan and nanochitosan are excellent natural adsorbents that can be modified to increase their efficiency and improve their basic properties. Chitosan-NPs composites can be classified as nanosorbents that meet the basic criteria for use in wastewater treatment^14^.

The use of microorganisms in the biosynthesis of nanoparticles is crucial due to the diversity of microbial by-products, both internally and externally, which have an essential role as reducing agents that convert elements from the ionic to the nanoform. Selenite (SeO_3_)^−2^, is highly soluble and biologically accessible and can be transformed by microorganisms to insoluble Se^0^^10^. In the process of selenium detoxification, microbial cells play a crucial role in the reduction of selenium oxyanions; some bacteria can create elemental selenium through this process using the oxidized form of selenium (selenite) as respiratory substrates. Because its quickly precipitates, selenium nanoform is mostly accessible to biological systems^15^. Numerous fungi, bacteria and plant extracts have been described for the bio-manufacturing of selenium nanoparticles. This microbial diversity and diversity in secondary metabolites leads to diversity in the size, shape, and coating agents of the resulting nanoparticles (and nanocomposites), and thus their effectiveness and properties. Hence, bacterial cell-free extracts can be used as biological catalysts in the biosynthesis of selenium nanoparticles (SeNPs), offering a non-toxic and green method for producing nanoparticles. Given their antibacterial properties and adsorptive properties, nanochitosan and the biosynthesized SeNPs could be useful in combination (or individually) as biofilter(s) for heavy metals removal and disinfection applications^16^.

Hence, the current study aimed to isolate and characterize selenium-resistant bacteria capable of green synthesis of selenium nanoparticles while maximizing culture conditions to improve selenium biosynthesis. In addition, developing a biosynthetic selenium nanocomposite with nanochitosan for use as a biofilter to remove mercury, AgNPs and pathogenic microbes from wastewater.

## Results and discussion

### Isolation of selenium-resistant *bacteria* for extracellular SeNPs biosynthesis

**Figure 1:**
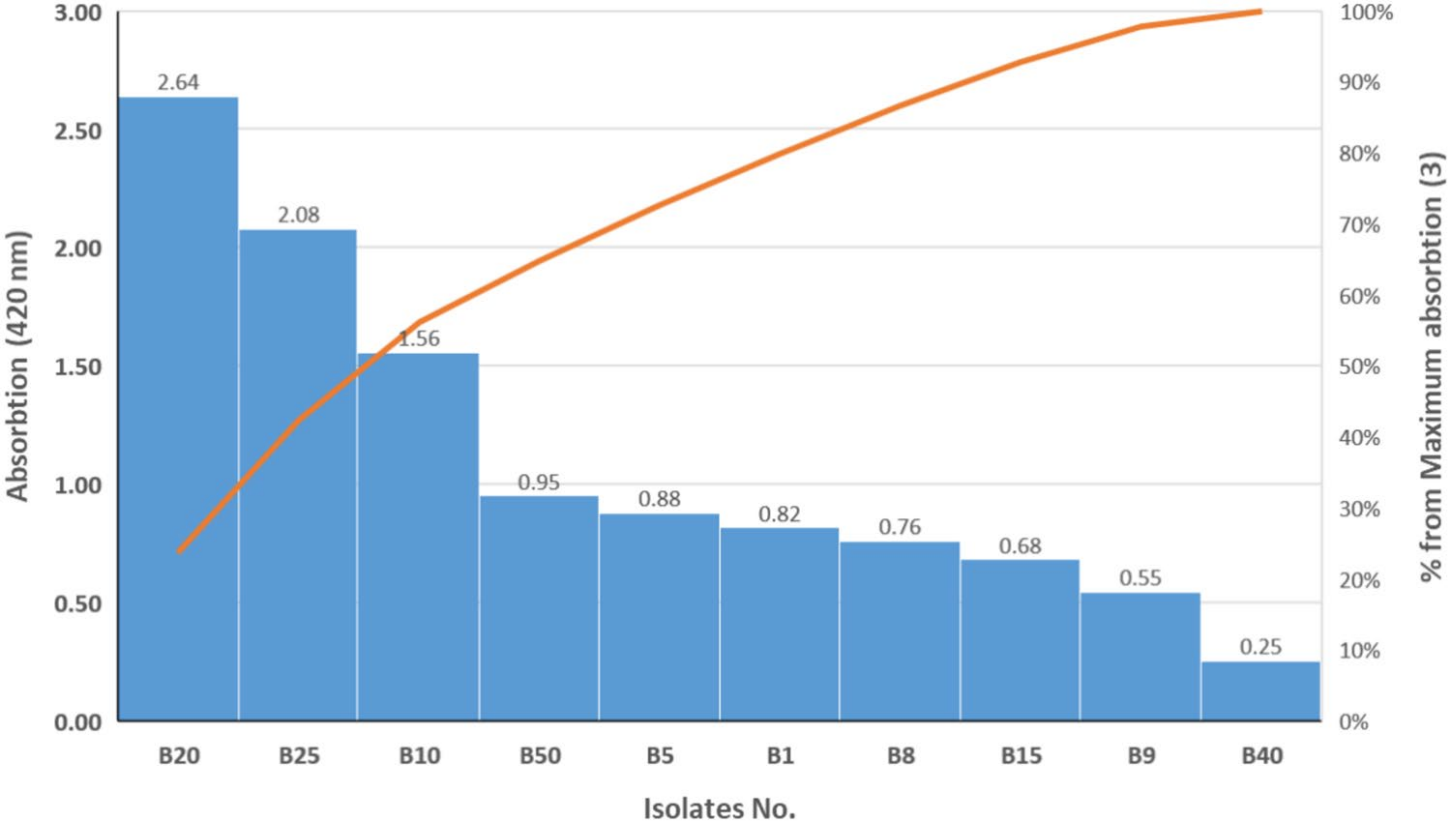
Screening of the positive selenium reduction isolates for their ability to extracellular SeNPs biosynthesis.

Selenium as an essential micronutrient plays an important role for plant, animal and human metabolic systems. SeNPs are gaining significant in medication field due to their anticancer and antibacterial properties and also can be applied for wastewater treatment^17^. They are biocompatible and less-toxic in comparison to their counterparts (selenite and selenate) as mentioned by Kumar and Prasad^9^. Thus, the aim of the study was focused on isolation of bacteria that have extracellular capability for reducing selenium ions outside cellular membranes and producing nanostructures. To realize this goal, 25 samples were collected from diverse Egyptian areas (10 samples of atmospheric soils and 15 of wastewater). The first indicator for the reduction activity of microbial community found in tested samples is appearing red or orange color in enrichment flasks^18^. After several successive re-enrichments, the microbial suspension from each sample was inoculated onto isolation medium plates. The grown colonies (red or orange color) were re-streaked onto the same medium for purification and/or confirmation of reduction efficacy. After isolation processes, 60 bacterial isolates observed the formation of red color on purification plates indicating selenium-reducing activity. Pure Se-resistant bacterial isolates were selected based on their extracellular biosynthesis of Se as a result of the reduction of surrounding Se ions (indicated by the red or orange colored region). These bacteria were subjected for screening their ability to produce extracellular metabolites containing reducing system by inoculation into MSM liquid medium containing selenium ions as inducer their reducing metabolites^19^. The cell-free supernatant as component rich in potential selenium reductants was mixed with serial concentrations of Se ions and the formed SeNPs were examined on a spectrophotometer (at 420 nm). Ten isolates had the ability to reduce increasing concentrations of sodium selenite up to 10 g/L and produced extracellular selenium nanoparticles. The reductive activity of these bacteria was demonstrated as shown in Fig. 1. As data noted in these results, bacterial isolate coded B20 was recorded as having high efficacy for the extracellular reduction of selenium ions and formation of selenium nanostructures. Therefore, this bacterium was selected for identification and further experiments.

### Molecular identification of the most efficient isolate

**Figure 2:**
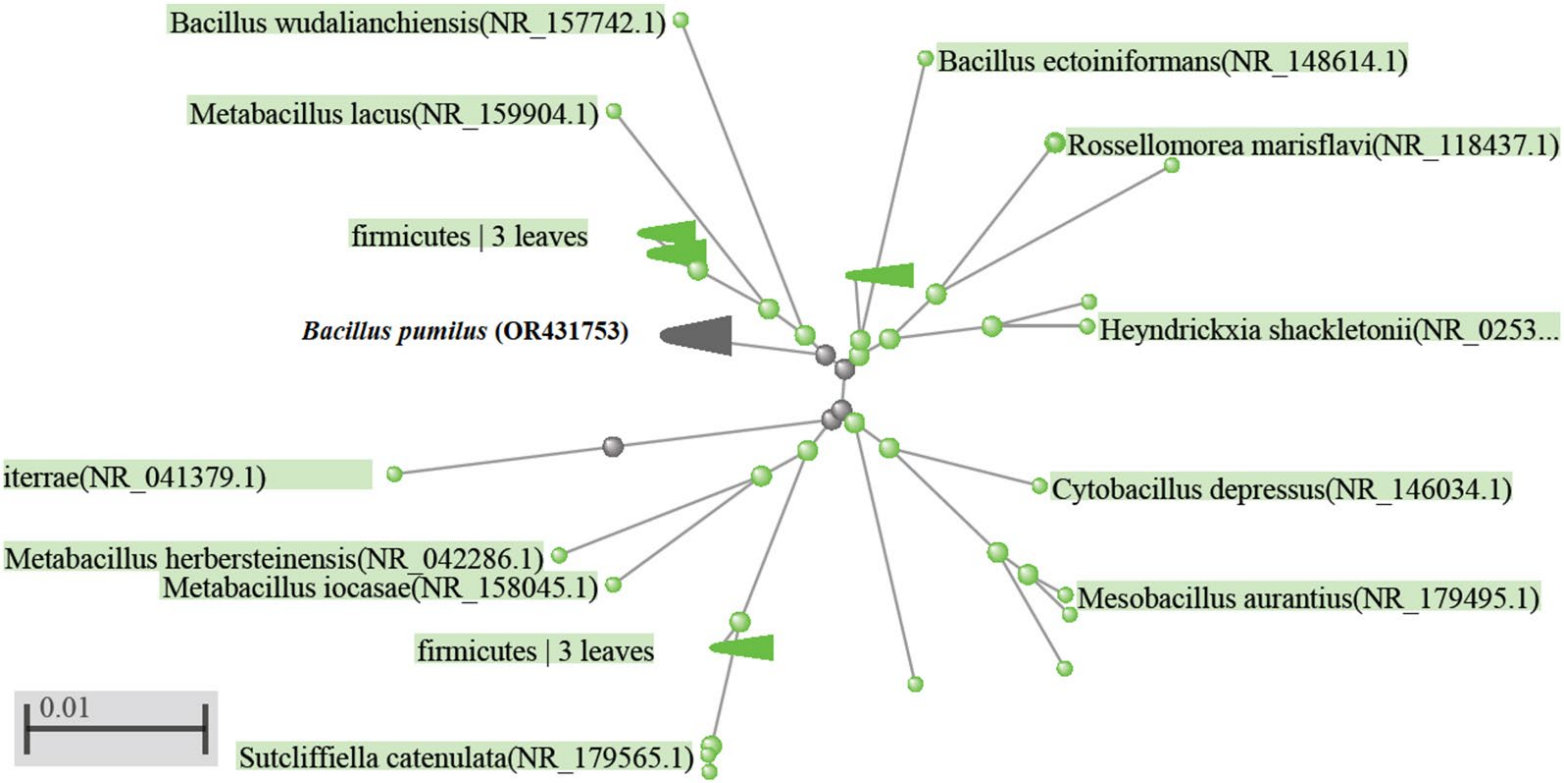
Phylogenetic tree for comparing the obtained bacterium (*B. pumilus* isolate OR431753) with related species on Genebank.

Bacterial isolate No. B20 was selected as the most active bacterium in producing high amounts of reducing agents for selenium reduction and fabrication of SeNPs. The isolate was Gram-positive rod bacilli, spore-forming with endospores, aerobic with peritrichous flagella. Its colonial morphology was variable (roughly circular, but with an irregular margin) with an opaque and off-white color. The previous characteristics led to the primary identify this bacterium as *Bacillus pumilus.* The identification procedures were confirmed by applying molecular biology techniques. The phylogenetic tree of bacterium isolate B20 as represented in Fig. 2 appeared closely relation between this isolate and *B. pumilus* in Genebank. Thus, the obtained isolate was identified as *B. pumilus* and deposited in Genebank under accession number of OR431753. *B. pumilus.* Previous research reported that this bacterium is commonly found in soil and used as an active ingredient in agricultural fungicides^20^.

### Enhancing the selenium reduction reaction and producing SeNPs

**Figure 3:**
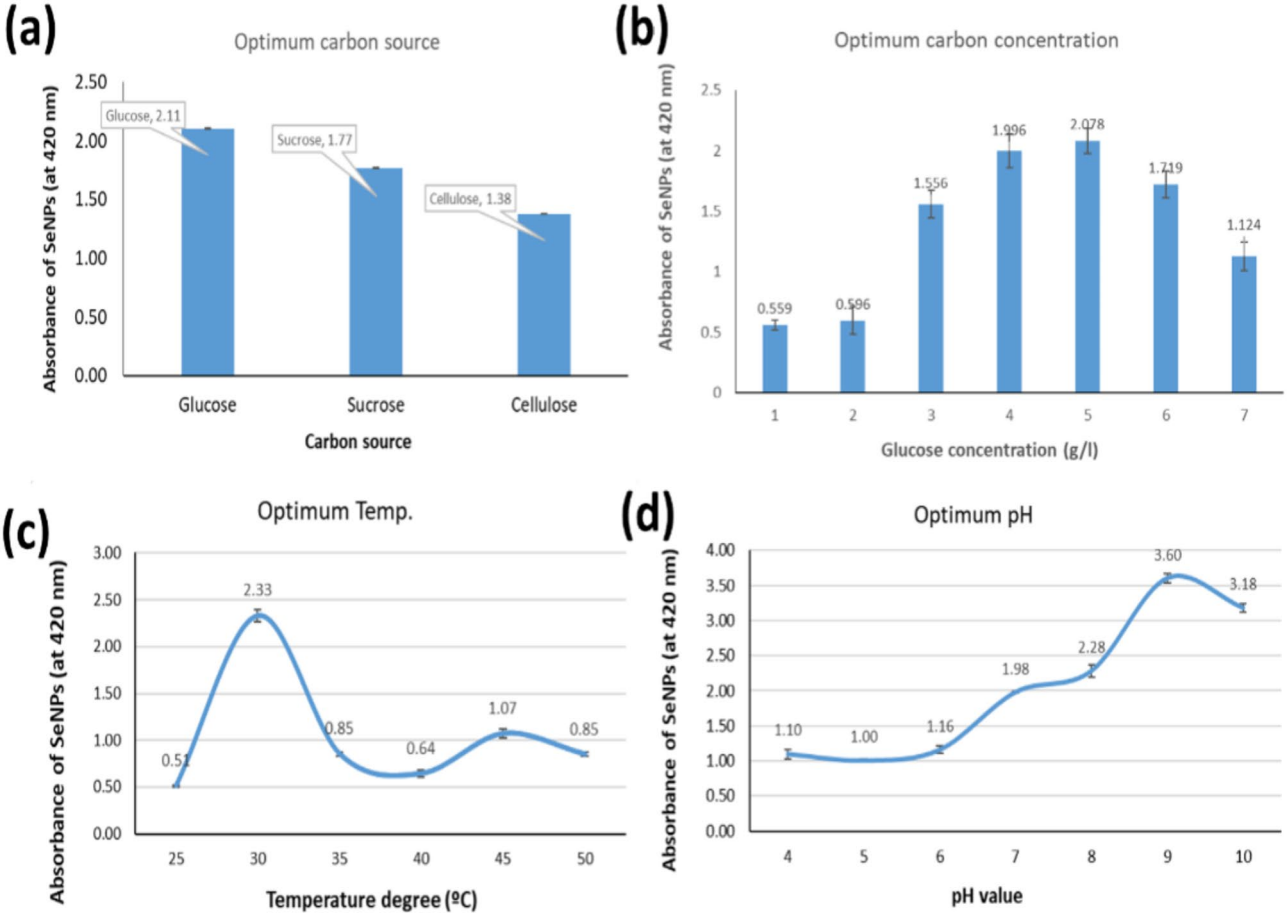
Optimization of selenium reduction reaction by *B. pumilus* isolate OR431753 under different carbon sources (**a**), glucose concentrations (**b**), temperature degrees (**c**), and pH values (**d**) for producing high yield of SeNPs.

To produce maximum amounts of SeNPs, it is important to excrete the high amounts of reducing agents that produced by *B. pumilus* isolate OR431753. The reducing agents as microbial metabolites are affected by cultural nutrients like carbon and nitrogen sources^21^. Therefore, in the present work, three carbon sources representing different sugar categories (mono, di, and polysaccharides) were studied to enhance reducing agents’ productivity. From the noted data, the monosaccharide glucose was the best one for producing a high absorbance of SeNPs (2.11 ± 0.003) as illustrated in Fig. 3a. Moreover, the optimized glucose concentration regarding extracellular SeNPs biosynthesis was 5 g/L (Fig. 3b). Glucose is an essential and easy carbon source for different heterotrophic microorganisms; its presence induces the production of either intra- or extracellular substances important for reducing elemental ions^22^. On the other hand, the environmental conditions during the process of bioreduction of mineral elements, particularly temperature and pH, affect the efficiency of its conversion to nanoform. Thus, six different temperatures and seven pH values were applied as controlled conditions to the bioreduction processes of extracellular SeNPs biosynthesis using *B. pumilus* cultural filtrate (Fig. 3c,d). Microbial reducing system like reducing enzymes (active proteins), phenolic compounds, polysaccharides and inactive proteins is needed especial environmental conditions to work^23^. From the data obtained in the current work, the optimized conditions for extracellular SeNPs biosynthesis using cultural filtrate of *B. pumilus* were due to incubation at 30 °C and pH 9.

### Production and characterizations of SeNPs

**Figure 4:**
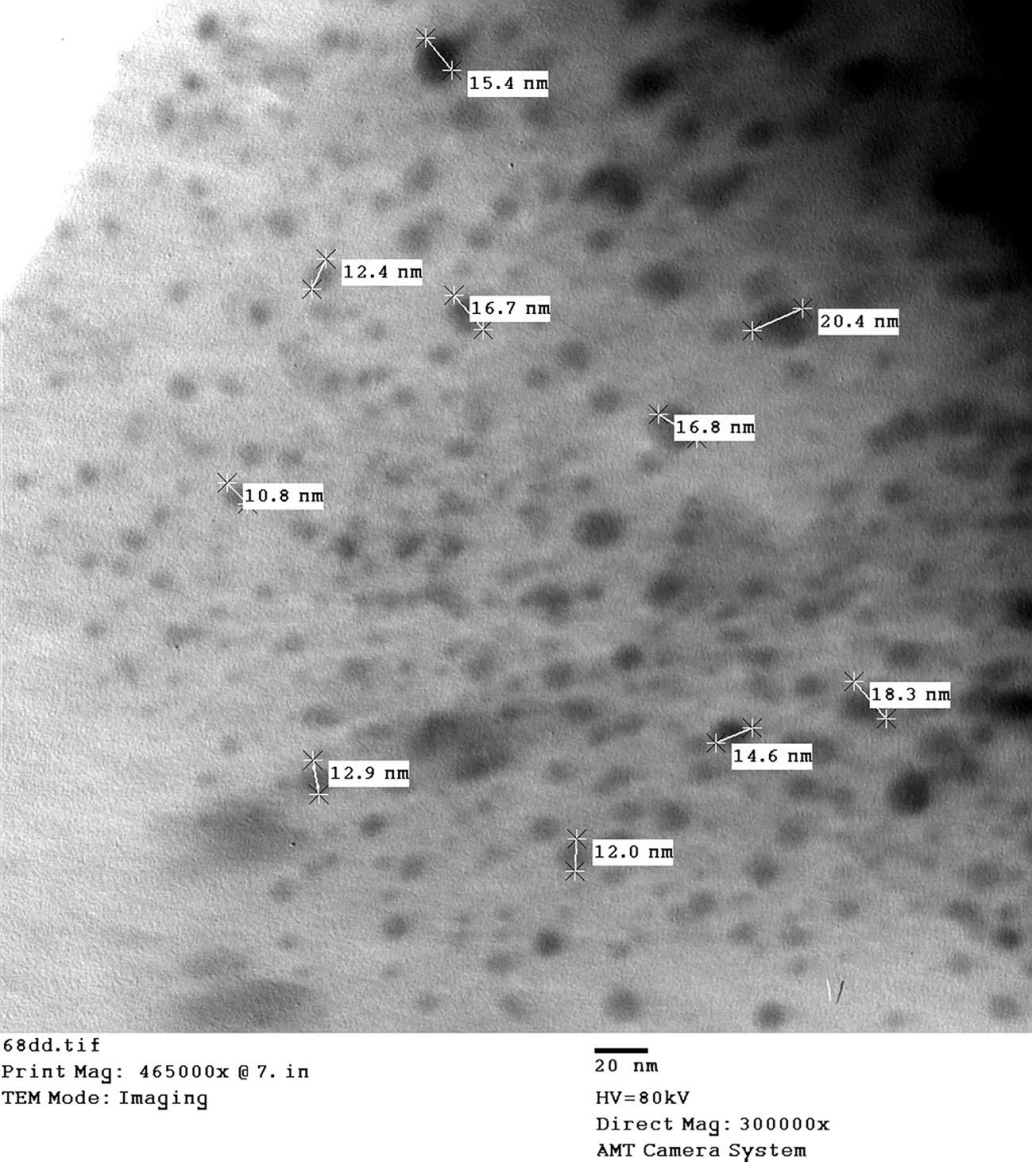
TEM image of SeNPs produced by *B. pumilus* isolate OR431753.

**Figure 5:**
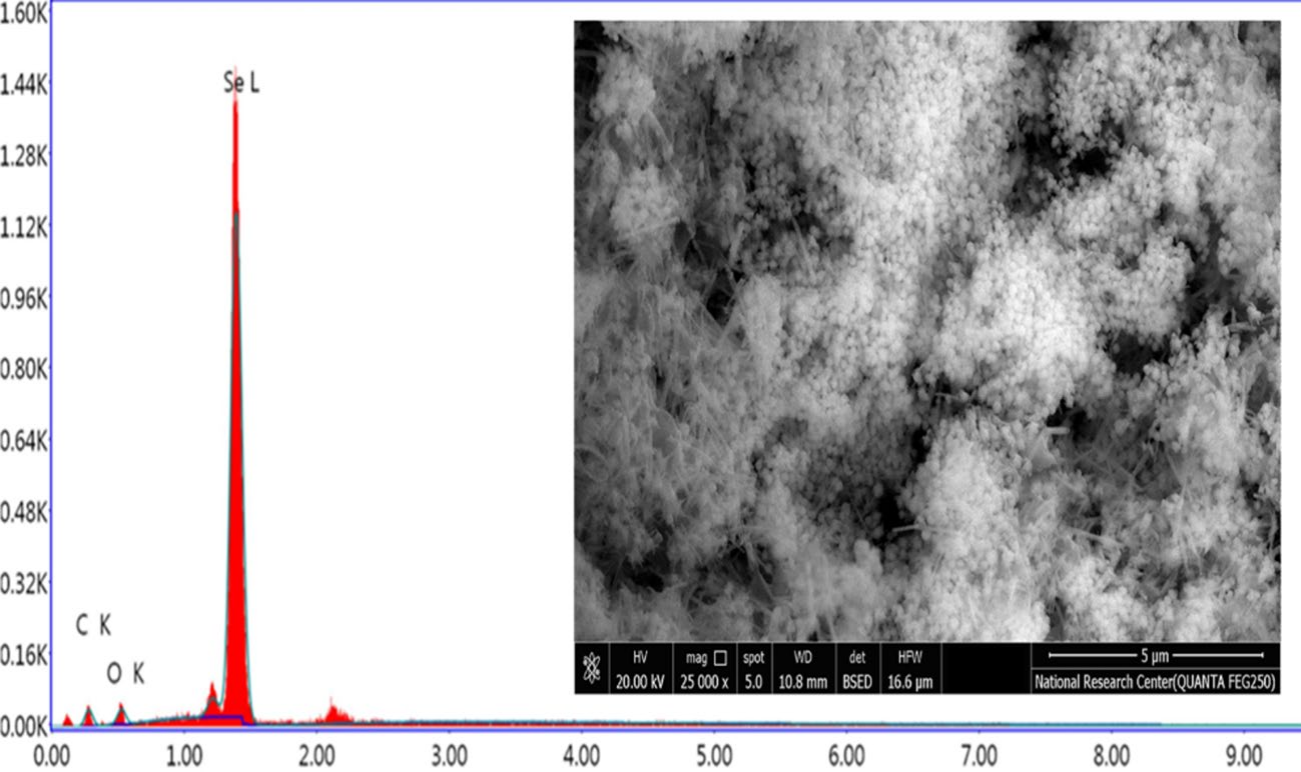
SEM–EDX characterization of SeNPs produced by *B. pumilus* isolate OR431753.

**Figure 6:**
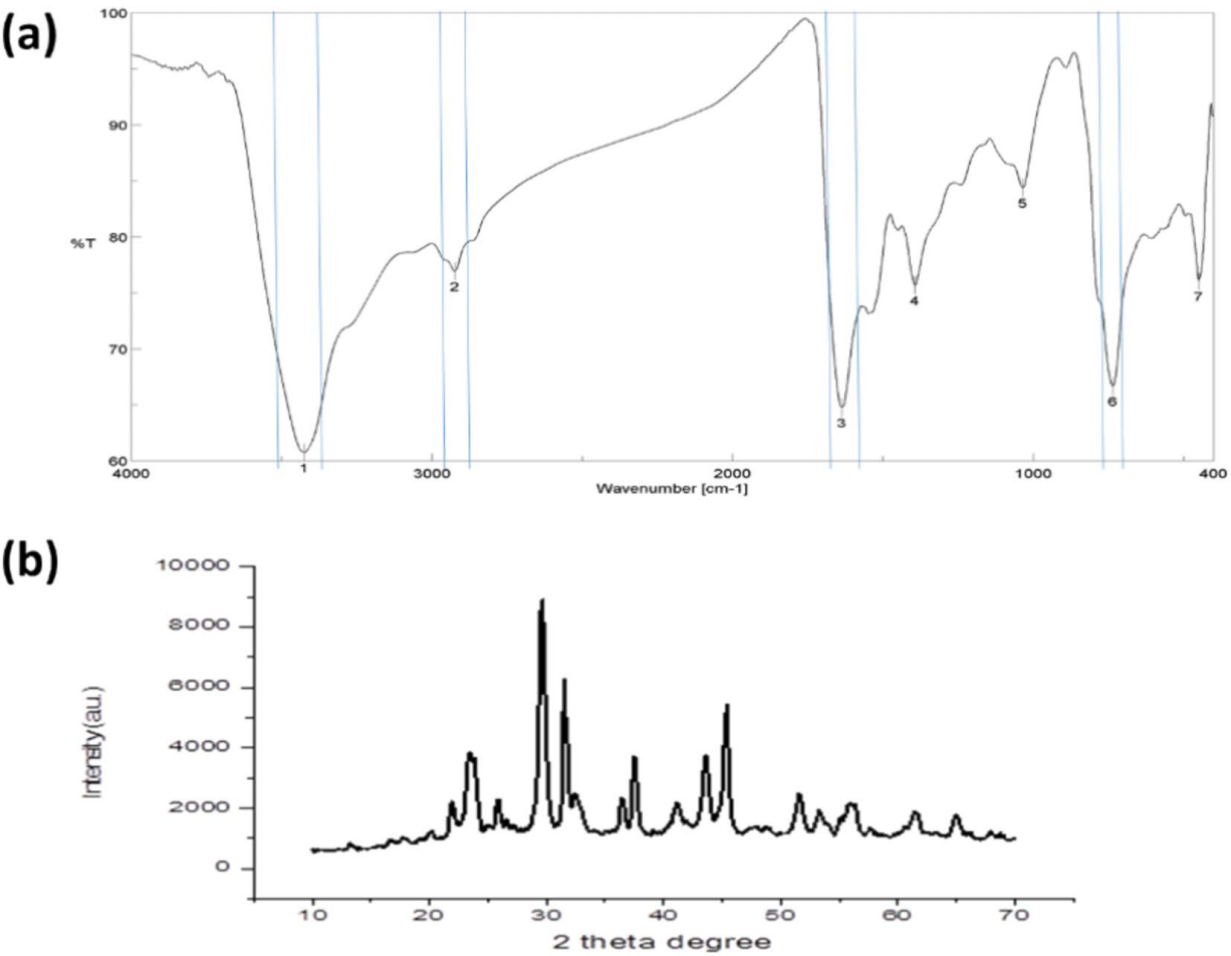
FTIR (**a**) and XRD (**b**) characterizations of SeNPs produced by *B. pumilus* isolate OR431753.

Optimal nutritional and environmental conditions were chosen to obtain high production of extracellular *B. pumilus*-SeNPs which were prepared for characterization. The produced SeNPs were characterized using a variety of tools, including TEM, SEM–EDX, FT-IR, and XRD. From TEM image as illustrated in Fig. 4, the size of the produced SeNPs was around 15 nm (ranged 10–20 nm) with spherical shape. The size of these nanoparticles is very important for biological applications especial that depend on bioactivity, due to its high surface area and easy to penetrate microbial cell wall^24^. The morphology of SeNPs as analyzed by SEM was uniform spherical particles (Fig. 5), and the elemental analyses revealed that purified Se was produced. Also, for determination of functional groups, data in Fig. 6a showed that the FT-IR peak at 3424.96 cm^−1^ was assigned to N–H and O–H elongating in the component polysaccharide and/or protein residues as previous mentioned by Blinov et al.^25^. The 2924.52 cm^−1^ illustrated C–H extending specifies the functional groups of aromatics, aldehydes and alkynes^26^. The peak obtained at 1636.3 cm^−1^ indicated the existence of some organic compounds like N–H bond of primary amines. From these data, it has been exposed that the cell-free supernatant produced by *B. pumilus* isolate OR431753 contained numerous biological compounds for the biosynthesis of SeNPs. Crystallinity versus morphs materials testing was performed using XRD analysis, from the graph produced by XRD (JCPDS card no. 36–1451), SeNPs were created at ordered structure and the arrangement of particles was uniform. Also, the peaks shown in Fig. 6b showed the small size of the produced nanoparticles, which was confirmed by both TEM and SEM analysis. The crystallinity of the bacterial-synthesized SeNPs was confirmed by XRD analysis.

### Characterization of reducing system causing SeNPs formation

The extracellular biosynthesis of NPs takes place through many reducing substances, that works individually or collectively to reduce the element from its ionic form to its nanoform. Many reducing agents have been reported *e.g.* proteins, sugars, phenolic compounds, flavonoids, and reductase enzymes. The type of microorganism and its growth conditions including nutrient types and concentrations influence the nature of excreted reducing agents^27,28^. Therefore, in the present work, several metabolites in the cell-free supernatant of *B. pumilus* were analyzed due to their potential role in the biosynthesis of SeNPs. The total content of extracellular carbohydrates, sugars, proteins, phenols and flavonoids was determined as a result of bacterial growth in a liquid MSM medium. The data represented in Table 1 illustrated that 25.23 mg/L of total carbohydrates were noted, half of that was polysaccharides (12.47 mg/L). Also, the total proteins (2220.73 µg/mL) were recorded as a high value. From that, we can see the primary metabolites played a vital role in the reduction and/or protection of SeNPs. Based on previous article, microbial metabolism was used to reduce selenium ions to SeNPs, also, generate organic ingredients like polysaccharides, proteins and lipids on the SeNPs surface for structure stabilization^29^. Polysaccharides as natural carbohydrates have rich active hydroxyl groups, and large specific surface areas, making them decorate the SeNPs surface by intermolecular non-covalent connectors^30^. The obtained results supported the discussion of reduction processes of Se ions and converting it to nanoforms, in addition to protect them from aggregation after its biosynthesis.

**Table 1:** Primary and secondary metabolites produced by *B. pumilus* isolate OR431753 through SeNPs formation.

Metabolic substances	Value
Total carbohydrate content (mg/L)	25.23
Polysaccharide content (mg/L)	12.47
Total protein content (µg/mL)	2220.73
Total phenolic compounds (µg/mL)	260.51
Total flavonoids (µg/mL)	1260.55
Total tannins (+ or −)	–

### Preparation of chitosan-SeNPs nanocomposite and evaluation its bioactivity

**Figure 7:**
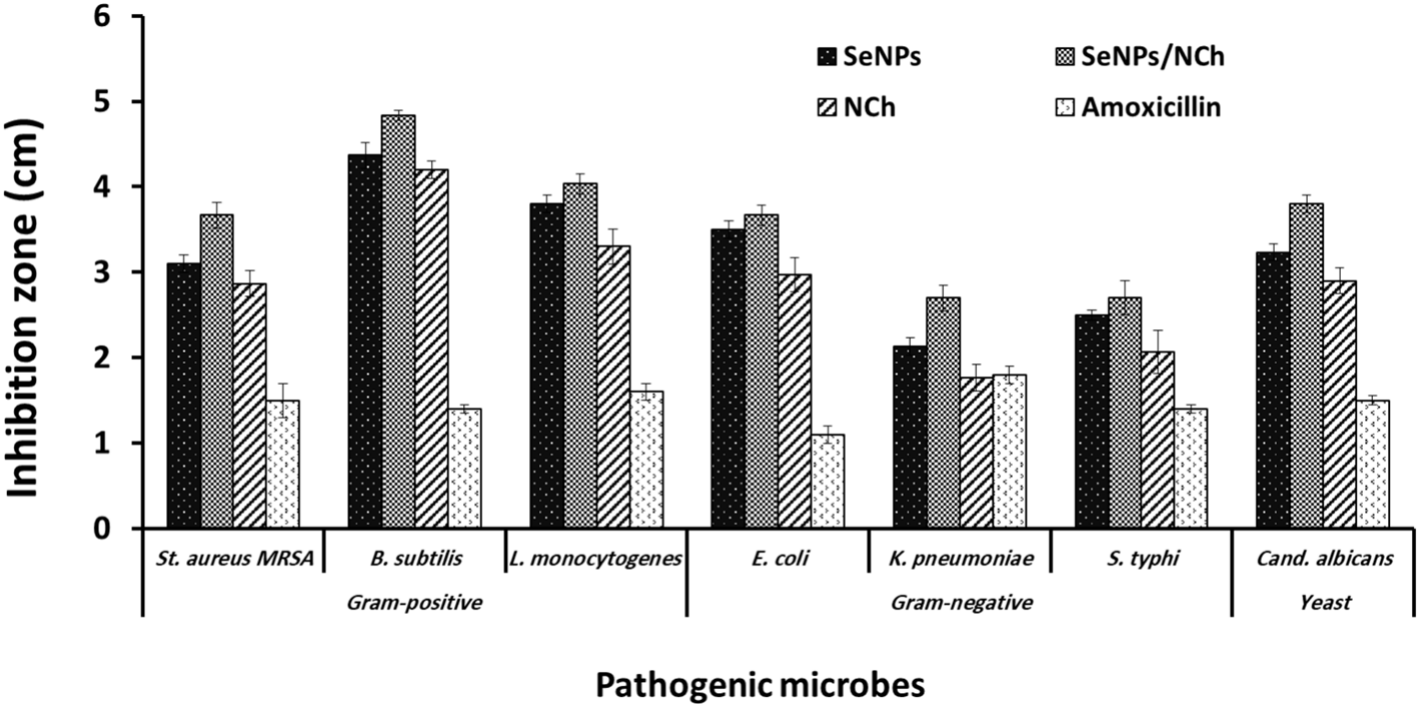
Antimicrobial properties of SeNPs and its nanocomposite with nanochitosan (SeNPs/NCh) comparing with Amoxicillin as standard antibiotic.

Chitosan extracted from shrimp waste has many biological activities such as anti-cancer, anti-bacterial and anti-inflammatory. In addition, this natural biopolymer has unique properties as an adsorbent with high biocompatibility and biodegradability^31^. Hence, it was used in the present work (in its nanoform) to support the biological properties of SeNPs, especially antibacterial activity and heavy metal removal from wastewater media. The antimicrobial activity of the prepared chitosan-SeNPs composites was evaluated against representative pathogens of both Gram-positive and Gram-negative bacteria (three each), as well as *Candida albicans* as a pathogenic yeast. From the data represented in Fig. 7, which is represented by the diameter of the inhibition zone against each pathogen on the agar plates, the prepared SeNPs had inhibitory activity against all pathogens tested. Moreover, it was observed to have more growth inhibiting activity of Gram-positive bacteria (about 3.2–4.5 cm) than Gram-negative bacteria (about 2.1–3.5 cm). The prepared chitosan-SeNPs nanocomposite, recorded enhanced antibacterial activity due to the addition of nanochitosan. Also, they had anti-candidal activity against pathogenic *Candida albicans* as the activity reached to 3.2 and 3.8 cm for SeNPs and SeNPs/NCh nanocomposite, respectively. Compared with Amoxicillin as a standard antibiotic (at a concentration of 250 mg), the prepared SeNPs and SeNPs/NCh nanocomposite have higher antimicrobial activity. The explanation for this superior antimicrobial activity may be due to the size of SeNPs, as they play a vital role in penetrating the microbial cell wall and damaging DNA and protein molecules. Consistently, Zhang et al.^32^ confirmed that the bio-SeNPs produced by *Providencia* sp. displayed greater antimicrobial activity than chem-SeNPs, and the activity was increased by decreasing the size of bio-SeNPs. Also, another research group reported that smaller nanoparticle sizes usually have relatively higher stability and antimicrobial action than larger sizes. This is because smaller nanoparticles have larger surface areas, providing superior reactivity and penetration of the bacterial cell wall^33^.

### Purification of water by instantaneous removal of aqueous mercury using syringe-nanocomposite-Se filter

**Figure 8:**
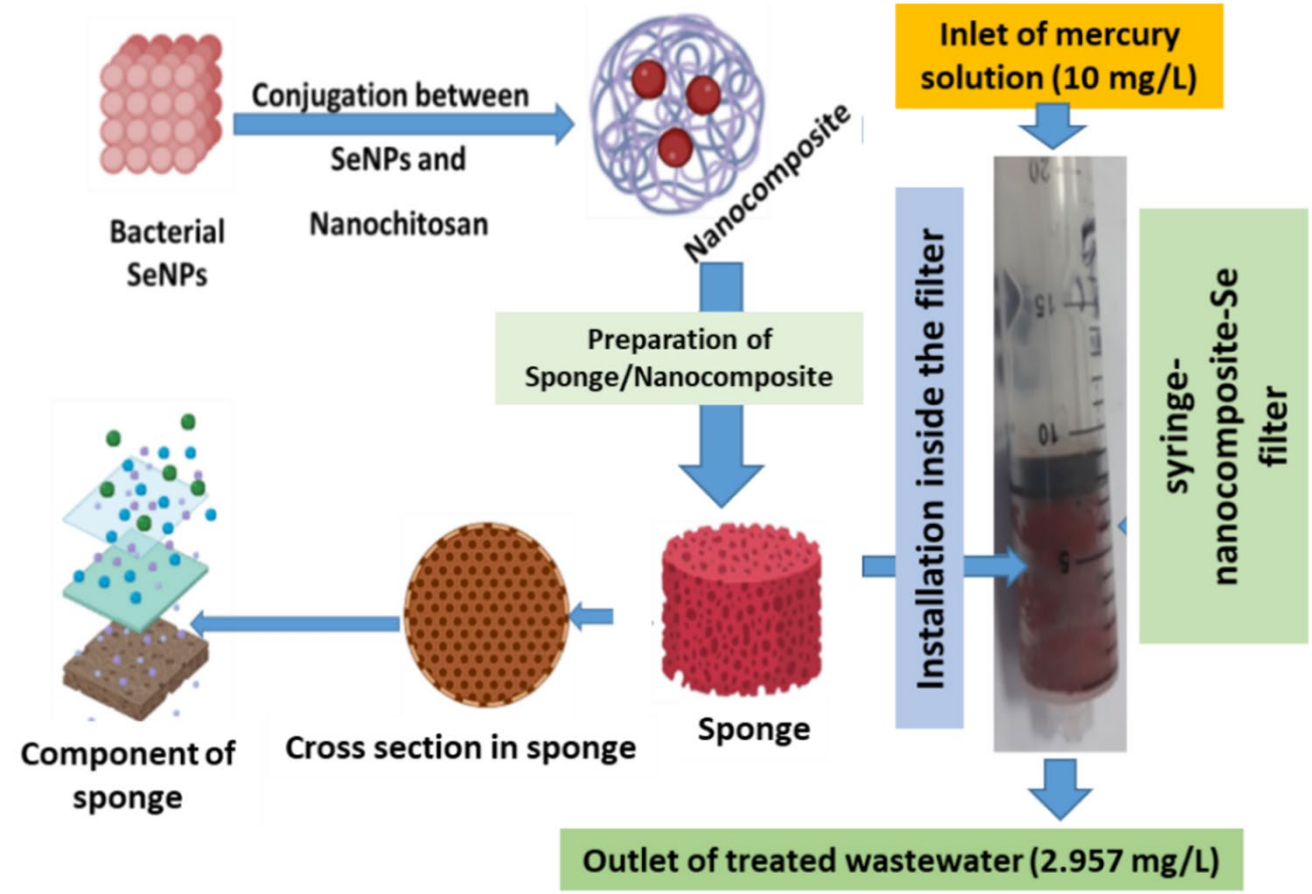
Purification of water contaminated by Hg^+2^ using syringe-nanocomposite-Se filter.

The designed syringe filter-Se nanocomposite, (a sponge filter containing SeNPs/NCh nanocomposite) (Fig. 8), was applied to purify mercury-contaminated water. To verify the effectiveness of this filter in removing mercury from aquatic media, synthetic wastewater containing 10 mg/L Hg^+2^ was passed through the filter. Measurement data after 10 s of processing the sample through the nanofilter indicated that it succeeded in removing about 99.97% (Fig. 8) compared to 10 and 89.85% for using the polyurethane sponge or SeNPs individually, respectively. The efficiency of SeNPs for mercury removal from contaminated water was associated in the current work with nanochitosan to develop its adsorption capacity. This is the first report for merging SeNPs adsorption capacity with polysaccharide (nanochitosan polymer). The current system for removing mercury ions from water is considered a development of the sponge system presented previously^34^, where they used a polyurethane sponge supported with nano-selenium to remove mercury ions. The mercury ions are considered as one of the most hazard toxic elements in aquatic environments, it is considered one of the top ten hazardous chemicals. Depending on the WHO Organization^35^ report, some facts should be considered; mercury naturally occurs and found in air, soil and water. Exposure to it in small amounts may cause serious health problems. It may have toxic properties on the digestive, nervous and immune systems, and has negative impacts on the skin, lungs, eyes and kidneys. People are mostly exposed to methylmercury (an organic compound produced from mercury by bacteria in a water environment) when they eat fish and shellfish. Therefore, it is necessary to remove this dangerous element (mercury) from water through environmentally safe technologies (which is our goal in this research). In the same context, many researchers have suggested that the mechanism of removing mercury from its aqueous solutions by nanocomposites is due to adsorption and reduction processes^36–38^.

### Application of SeNPs/NCh -nanocomposite filter in wastewater bioremediation for AgNPs removal and pathogen disinfection

**Figure 9:**
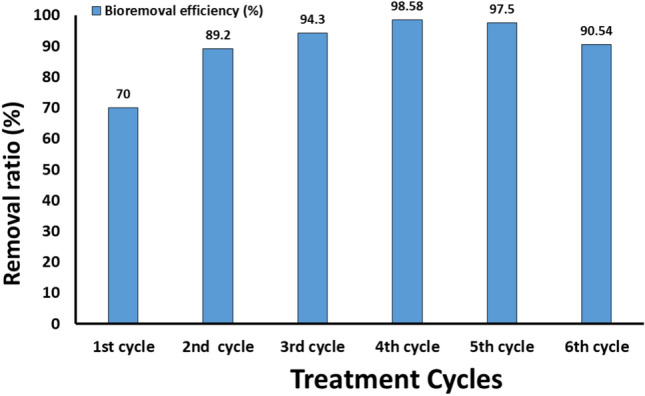
Bioremoval efficiency of silver nanoparticles by using syringe-nanocomposite-Se filter.

The spread of applications of nanomaterials in almost all sectors (especially nanosilver) leads to an increase in the chances of their waste spreading to wastewater, soil, water bodies, and thus plants, animals, and humans^5,39^. This leads to the need to treat contaminated sites through effective and environmentally safe technology. Hence, the nanocomposite-Se syringe filter was applied to treat industrial wastewater containing nanosilver or pathogenic microbes and the results are shown in Fig. 9. The effectiveness of bioremoval reached about 99% after passing the wastewater four cycles of five minutes each through the designed SeNPs/NCh-nanocomposite filter. In case of disinfection of pathogenic microbes, the reduction in microbial counts reached to 98.96%. The obtained data stated that the applied filter had high efficiency for the remediation of wastewater containing silver nanoparticles or pathogenic microbes. Bioremoval efficacy may be returned to adsorption capacity of nanochitosan as polysaccharide polymer, which supported SeNPs in purification of water and wastewater. Therefore, this filter may be a suitable solution for the biological treatment of municipal and/or industrial wastewater to remove many heavy metals and pathogens present in it. In this line, Shalaby et al.^5^ published the first report in bioremoval of some nanomaterials like silver, zinc and selenium from their aqueous solutions by an efficacy of 78%. There is a lack of research articles that deal with the biological treatment of NPs-contaminated wastewater. Thus, the current work is considered a step in using biosynthesized SeNPs-chitosan nanocomposite as an antimicrobial and adsorbent filter for metals and nanopollutants.

## Materials and methods

### Materials, reagents, and media

Anthrone, folin-ciocalteu reagent, Gallic acid, Tripolyphosphate and chitosan were purchased from Sigma-Aldrich, VWR chemicals and Merk, India. Yeast extract, Nutrient broth and Nutrient agar media were obtained from HiMedia Leading BioSciences Company, India. DNA extraction chemicals and PCR reagents were purchased from Sigma-Aldrich, India. QIAquick Gel Extraction Kit was obtained from QIAGEN Company, USA. All chemicals and reagents used were of analytical grade and purchased by lab suppliers from Sigma-Aldrich, VWR chemicals and Merk, India.

### Isolation of selenium-resistant *bacteria* for extracellular reducing selenium ions

The isolation of Se-resistant bacteria was performed following enrichment culture technique on liquid medium supplemented with selenium. Briefly, 10 g of rhizosphere soils (10 samples) or 10 mL of wastewater samples (15 samples) were individually placed in 250-mL Erlenmeyer flasks containing 90 mL enrichment medium and incubated under shaking conditions (100 rpm at 30 ± 2°C) for overnight. The enrichment medium (EM) consist of 50 mg/L of Se ions as 0.5 g/L of Na_2_SeO_3_·5H_2_O and 1 g/L yeast extract. From this bacterial culture mixture, multiple transfers of 2.5% of the old cultures were enriched into new enrichment medium (EM), and incubated at 30 ± 2 °C and 100 rpm for three days. The activity of bacteria was visually observed (formation of red or orange color of reduced Se ions in enrichment medium). After three successive EM cultures, a loopful of enriched culture was streaked onto the surface of Mineral Salts Medium (MSM) agar plates supplemented with Se and incubated at 30 ± 2 °C for 5 days. The Se-MSM solid medium contains (g/L); 2.9 of glucose, 1 of ammonium sulfate, 0.1 of NaCl, 1 of K_2_HPO_4_, 0.5 of KH_2_PO_4_, 0.2 of MgSO_4_, 0.5 of yeast extract and 18 of agar^10^ as well as 50 mg/L Se ions. The bacterial colonies surrounded by red or orange zones were selected and transferred individually to new plates for confirmation before purification on new Se-MSM agar plates. Pure bacterial isolates were selected based on their extracellular reduction of Se ions (as red or orange zone on agar plates). The bacterial isolates were maintained on MSM agar slants containing 50 mg/L Se ions, reserved at 4 °C in a refrigerator and sub-cultured every three months.

### Screening the obtained isolates in reducing Se ions at high concentrations

Firstly, the obtained isolates (60 bacterial isolates) were inoculated into liquid MSM medium (10 mL) supplemented with 50 mg/L Se ions and incubated at 30 ± 2 °C for 2 days. The growing cultures were centrifuged at 1509 xg for 7 min and the supernatant was individually collected. Each supernatant was added to an equal volume of sodium selenite solution (0.5 g/L) and incubated at static conditions for overnight. The formation of red or orange color was considered as indicate for positive results of selenium ions extracellular reduction. The cell-free supernatant of positive isolates was subjected to a gradual increase in concentrations of sodium selenite from 1 to 10 g/L. After overnight incubation of the mixtures, the mixture was sonicated and then centrifuged at 6037 xg for 10 min and the precipitates were washed three times by deionized water and then suspended in a fixed volume of deionized water. The suspended reduced selenium was detected by measuring the absorption at 420 nm. The occurrence of SeNPs in the solution was confirmed by the feedback's color shift from yellow to brilliant red^10^.

### Genotypic identification of the most efficient isolate

One bacterial isolate was submitted to genotypic identification based on its role in the reduction of selenium to its nanoform higher than other isolates. The total genomic DNA of bacterial isolate was extracted using the lysozyme-sucrose technique which was detailed previously^40^. The 16S rDNA as a universal gene for bacterial molecular identification of the selected bacterium was amplified using 2 primers, the forward primer was 16RW01 (5'- AACTGGAGGAAGGTGGGGAT-3') and the reverse one was 16DG74 (5'- AGGAGGTGATCCAACCGCA-3'). The purification of PCR product was performed by QIAquick Gel Extraction Kit (QIAGEN, USA) after running on agarose gel before sequencing of 16S rDNA fragments. The bacterial molecular sequence was compared with known sequences in the GeneBank nucleotide databases and identified as the nearest phylogenetic neighbor with the highest similarity percent^41^.

### Enhancing the selenium reduction reaction and producing SeNPs

The enhancement of extracellular selenium reduction capacity by cell-free cultural filtrate of the selected bacterium was examined after changing the carbon sources used during growth. The monosaccharide glucose (the original carbon source of MSM medium) was replaced by either sucrose as a disaccharide or cellulose as a polysaccharide as a carbon source. The carbohydrates were added to the medium based on their carbon content (calculated based on 2.6 g/L glucose). In addition, different concentrations of glucose *i.e.* 1, 2, 3, 4, 5, 6, and 7 g/L were also examined for cultivation of the selected bacterium for SeNPs biosynthesis. 250-mL conical flasks containing 100 mL of MSM medium with different carbon sources supplemented with 50 mg/L Se ions were prepared in triplicate, sterilized and inoculated by the isolated bacterium. The cultures after 3 d of incubation were centrifuged at 1509 xg for 7 min and the supernatant from each carbon source was individually collected. Each supernatant was added to an equal volume of sodium selenite solution (5 g/L) and incubated at static conditions for overnight. The produced SeNPs after centrifugation were collected, washed and re-suspended in a fixed volume of deionized water and measured at 420 nm.

The incubation temperature degree is very important factor for making biological reduction systems^42^. The effect of reduction reaction incubation temperature degrees on the reduction activity of the selected bacterial isolate was studied using MSM medium with glucose as a carbon source and pH 7. The collected supernatant was mixed with an equal volume of sodium selenite solution (5 g/L) and incubated at static conditions overnight at different incubation temperatures *i.e.* 25, 30, 35, 40, 45, and 50 °C. The produced SeNPs were collected by centrifugation, washed and re-suspended in a fixed volume of deionized water and measured at 420 nm.

The effect of pH values on the reduction system activity of the selected bacterial isolate was studied using MSM medium with glucose as a carbon source at 30 °C and pH 7. The collected supernatant was mixed with an equal volume of sodium selenite solution (5 g/L) and incubated at static conditions for overnight with different pH values *i.e.* 4, 5, 6, 7, 8, 9, and 10. The produced SeNPs were collected by centrifugation, washed and re-suspended in a fixed volume of deionized water and measured spectrophotometrically at 420 nm.

### Production and characterizations of SeNPs

SeNPs at powder form were obtained under optimum production conditions for characterization and application experiments. The SEM–EDX measurements were done on the recovered selenium nanoparticles powder at a 3kV accelerated voltage as well as the elemental analysis was conducted on the scanning electron microscope (Supra-55, Carl Zeiss AG, Germany) equipped with energy dispersive spectrometer^43^. The transmission electron microscopy (TEM) images were performed to determine the morphology and average size of SeNPs (TEM–JEM-2100 F, JEOL, Japan) . For this purpose, one drop of the suspended SeNPs loaded on the copper grid after sonication was allowed to evaporate completely before investigation^44,45^. Fourier-transform infrared spectroscopy (FTIR) was performed to detect biological functional groups produced related to reducing and/or capping agents. All spectra ranging between wave-numbers 4000 and 400 cm^−1^ were recorded with Fourier transform infrared spectroscopy (VERTEX 80 spectrophotometer, Bruker, Germany). For data processing, the software OPUS (version 2.2) was used for sample identification (Bruker). All spectra were baseline corrected and vector normalized. The spectral resolution was set at 4 cm^−1^ ^46^. For crystallinity of the obtained SeNPs, it was examined using Powder X-Ray Diffractometer (Model-D2 Phaser 2nd Gen, Bruker, Germany) using JCPDS card no. 36–1451. The biosynthesized SeNPs colloid deposited on a glass slide to form a thin film was performed on the X’Pert-Pro MPD x-ray diffractometer equipped using the Cu anode radiation of wavelength 1.54060 A. The scattering angle range was 10°–70°. The 2θ peaks were noted to confirm the presence of the nanoparticles^47^.

### Characterization of potential bio-reducing agents for SeNPs synthesis

The extracellular bacterial metabolites were analyzed to determine the main bioactive and reducing agents used for SeNPs production. Total carbohydrates, total proteins, polysaccharides, total phenols, and total flavonoids were measured in the cell-free supernatant. For determination of total carbohydrates, 0.1 mL of supernatant was added into a test tube containing 0.9 mL dH_2_O. The tube was immersed in ice and added 4 mL of cold anthrone, and then transferred to boiling for 8 min. After cooling rapidly, the developed blue-green color was measured by a spectrophotometer at 620 nm.

In case of total protein determination, 1 mL of supernatant was pipetted with 0.9 mL of reagent A (0.2% potassium sodium tartarate and 10% sodium carbonate) and incubated at 50 °C for 10 min in a water bath. After cooling, 0.1 mL of reagent B (2% potassium sodium tartarate and 1% copper sulphate) was added to the mixture, mixed well and then allowed to stand for 10 min. Three mL of reagent C (tenfold diluted folin-ciocalteu reagent) was added rapidly with vigorous mixing. The tubes were incubated at 50 °C in a water bath for 10 min and then measured at 650 nm after cooling.

The total flavonoids content was determined based on a method described by Eweys et al.^48^ with few changes. In brief, 0.5 mL of supernatant diluted with 2.5 mL of distilled water was mixed with 1 mL of 1 M potassium acetate and 1 mL of aluminum chloride (10%). The total flavonoids’ content was assessed by measuring the reaction absorbance at 415 nm using a UV–Vis spectrophotometer using quercetin as a standard. The total tannins were also measured by adding a few drops (3–5) of 0.1% FeCl_3_ to 5 mL of supernatant and the mix was changed to brownish green or blue-black color as an indicator for the tannins presence.

The total phenolic content (TPC) was examined according to Darwesh et al.^49^ with minor modifications. A 0.5 mL of supernatant was mixed with 0.5 mL of 10% Folin-Ciocalteu’s reagent diluted in 13 mL of dH_2_O before adding 2.5 mL of 7% Na_2_CO_3_ solution and mixing. The mixture was incubated for 2 h at room temperature in the dark. The absorbance was measured at 760 nm and the TPC was calculated using Gallic acid as a standard. For determination of polysaccharides, a standard curve of glucose was prepared using stock solution 1 mg/mL and its serial dilutions. For samples and standards, 1 mL of phenol solution (5%) was added to 1 mL of sample or standard solution followed by 5 mL of concentrated H_2_SO_4_ and incubated at room temperature for 10 min. The absorbance was measured against a blank at 488 nm.

### Preparation of nanocomposite chitosan-SeNPs

The nanocomposite from the produced SeNPs and chitosan (Ch) was prepared using ionic gelation technique^50^. After the solubilization of chitosan, the nanocomposite was developed after mixing a ratio (10% w/v) of Se/Ch and addition of 1% Tripolyphosphate (TPP) drop wise to prepare nanochitosan (NCh) with stirring until pellets precipitates. The formed pellets of SeNPs/NCh were washed several times by deionized water and one time by 50% ethanol and dried at 50°C.

### Antimicrobial activity of SeNPs and SeNPs/NCh

The antimicrobial activity of SeNPs and SeNPs/NCh was assessed by the well diffusion method according to Khaled et al.^51^ with slight modification. Gram-positive pathogens (MRSA and *Bacillus subtilis* and *Listeria monocytogenes*), Gram-negative pathogens (*Escherichia coli*, *Klebsiella pneumonia* and *Salmonella Typhi*) and *Candida albicans*, purchased from American Type Culture Collection were used as a model of pathogenic strains to evaluate the antimicrobial activity of tested samples. The nutrient agar plates were prepared and 70 μL of each activated pathogenic culture was spread by sterile swab. Wells of 6 mm diameter were made and 100 μL containing 50 mg/mL of SeNPs or SeNPs/NCh were added individually. The plates were incubated at 37 °C for overnight and the zones of inhibition formed surrounding the wells were noted. The results were compared with a set of standard antibiotic; Amoxicillin 250 mg.

### Design and operation of syringe-nanocomposite filter as a bioreactor for wastewater treatment

The bioreactor-based syringe-nanocomposite filter was designed for bioremediation of wastewater containing mercury, silver or pathogenic microorganisms. Polyurethane (PU) foam was prepared in a plastic bottle using the one-shot method as described by Tanaka, and his coworkers^52^. SeNPs/NCh were added to the polyol material (Diethylene triamine) at a concentration of 0.1% and mixed well before adding methane diphenyl isocyanate. After growing of PU sponge, the sponge was transferred into 20 mL syringe filter. The control filter was prepared also at the same conditions without adding SeNPs/NCh to the mixture.

### Purification of water by instantaneous removal of aqueous mercury using syringe-nanocomposite-Se filter

For the removal behavior of Hg from water, Hg solution (20 mL) with a concentration of 10 mg/L was passed through the syringe filter of the nanocomposite-Se-PU sponge. Firstly, the plunger was removed and the Hg solution was added to the syringe filter. The plunger was pressed slowly until all the solution was absorbed by the sponge and left 10 min for full absorption. After that, the bottom stopper was opened and the samples were collected and then analyzed for their contents of mercury using an inductively coupled plasma optical emission spectrophotometer (ICP-OES). A certified reference stock standard solution of Hg (1000 mg/L) purchased from "Merck" was used in this experiment (as a standard or for sample preparation). The samples were injected into ICP-OES through an ultrasonic nebulizer which converts liquid samples to very fine aerosol. The aerosolized sample was carried into the center of the plasma by the inner nebulizer argon flow.

### Bioremediation of wastewater containing nanosilver or pathogenic microbes using syringe-nanocomposite-Se filter

The synthetic wastewater was prepared by a known concentration of silver nanoparticles (AgNPs, 200 mg/L) suspended in dH_2_O. Twenty mL of AgNPs was passed through the syringe filter and the samples were injected into ICP-OES to determine Ag concentration. The removal efficacy was calculated against the control (sample before passing the syringe filter). For the reduction of microbial counts in wastewater, 20 mL of wastewater containing 10^8^ cfu/mL microbial counts was passed through the syringe filter. The collected samples were serially diluted and counted on nutrient agar plates. The efficacy of the bioreactor in reduction of microbial counts was calculated based on counting the microbial numbers before and after passing from the biofilter ^46^.

### Ethics approval and consent to participate

Experimental procedures comply with relevant institutional, national, and international guidelines and legislation. The manuscript did not have plant, animal, and human trials, so, the ethics approval was not applicable.

## Data Availability

Sequence data that support the findings of this study have been deposited in the National Center for Biotechnology Information with the primary accession code OR431753.

## Conclusions

The bacterium *B. pumilus* OR431753 can be used for the biosynthesis of extracellular SeNPs by reducing selenium ions with the potential to improve selenium nanoparticle production by controlling culture conditions (carbon source, pH, and temperature). The antimicrobial properties and adsorption ability of both biosynthesized SeNPs and nanochitosan can be exploited as a biofilter to remove some metal and nano-pollutants and reduce microbial contamination of water and wastewater. The resulting SeNPs/NCh nanocomposite has proven its efficiency as a filter for treating water or wastewater contaminated with mercury residues or AgNPs (with up to 99% efficiency for each). The positive results obtained require further studies on the long-term stability and efficiency of biosynthesized SeNPs/NCh nanocomposites as a candidate for several environmental pollutants.
